# Combined PCR and MAT improves the early diagnosis of the biphasic illness leptospirosis

**DOI:** 10.1371/journal.pone.0239069

**Published:** 2020-09-11

**Authors:** Noraini Philip, Norliza Bahtiar Affendy, Siti Norbaya Masri, Muhamad Yazli Yuhana, Leslie Thian Lung Than, Zamberi Sekawi, Vasantha Kumari Neela

**Affiliations:** 1 Department of Medical Microbiology and Parasitology, Faculty of Medicine and Health Sciences, Universiti Putra Malaysia, Serdang, Selangor, Malaysia; 2 Infectious Diseases Unit, Internal Medicine Department, Universiti Teknologi MARA, Sungai Buloh, Selangor, Malaysia; Bharathidasan University, INDIA

## Abstract

The diagnosis of leptospirosis remains a challenge due to its non-specific symptoms and the biphasic nature of the illness. A comprehensive diagnosis that includes both molecular (polymerase chain reaction (PCR)) and serology is vital for early detection of leptospirosis and to avoid misdiagnosis. However, not all samples could be subjected to both tests (serology and molecular) due to budget limitation, infrastructure, and technical expertise at least in resource-limited countries. We evaluated the usefulness of testing the clinically suspected leptospirosis cases with both techniques on all samples collected from the patients on the day of admission. Among the 165 patient’s blood/serum samples tested (from three hospitals in Central Malaysia), 43 (26%) showed positivity by microscopic agglutination test (MAT), 63 (38%) by PCR, while 14 (8%) were positive by both MAT and PCR. For PCR, we tested two molecular targets (*lipL32* by qPCR and 16S rDNA or *rrs* by nested PCR) and detected *lipL32* in 47 (29%) and *rrs* gene in 63 (38%) patients. The use of more than one target gene for PCR increased the detection rates. Hence, a highly sensitive multiplex PCR targeting more than one diagnostic marker is recommended for the early detection of *Leptospira* in suspected patients. When the frequencies for positivity detected either by MAT or PCR combined, leptospirosis was diagnosed in a total of 92 (56%) patients, a higher frequency compared to when samples were only tested by a single method (MAT or PCR). The results from this study suggest the inclusion of both serology and molecular methods for every first sample irrespective of the days post-onset of symptoms (DPO) collected from patients for early diagnosis of leptospirosis.

## Introduction

Leptospirosis, locally known as the rat-urine disease, is a re-emerging infectious zoonotic disease with a worldwide distribution [1–3]. In Malaysia, leptospirosis has been reported as early as in the 1920s among civilians and military troops [4]. To date, at least 41,736 cases with 502 deaths have been recorded corresponding to an average of 16 cases per 100,000 population annually (data from Ministry of Health, Malaysia) [5]. Because of its global distribution, with greater incidence in resource-poor populations and countries, leptospirosis gains more attention [1–3, 6, 7]. *Leptospira*, the etiologic agent of this disease not only affects humans but also infects and habituates a wide range of animals. Humans get infected either through direct contact with the infected or reservoir animals or indirect exposure to the contaminated environments. Leptospirosis, which was previously understood as an occupational disease [8, 9] is now frequently encountered in those involved in water and outdoor activities.

During recent years, there has been a steady progression in the understanding of the epidemiology and pathogenesis of leptospirosis. Diagnostic methods and tools were also extensively been studied and developed. Detection of *Leptospira* based on visualization (microscopy examination) and culture had been considered ineffective as microscopy could not detect the low number of *Leptospira* in clinical specimens [10, 11] with its low specificity [12] and the need for long incubation period (minimum four weeks) for culture. Serological kits such as enzyme-linked immunosorbent assay (ELISA) and rapid tests are also available, however, most suffer from low sensitivity and specificity [13–16]. Currently, the confirmatory methods for diagnosis of leptospirosis include the serology based microscopic agglutination test (MAT) and molecular-based polymerase-chain reaction (PCR). MAT is laborious, requires live cultures, has a very subjective interpretation, and often needs paired serum for confirmation, while PCR needs expensive reagents, technical expertise, and equipment such as thermal cycler.

In Malaysia or most resource-limited countries, any clinically suspected leptospirosis cases are subjected to rapid tests for immunoglobulin M (IgM) (in the hospital) or ELISA, and those positive or with inconclusive results are then subjected to MAT. PCR is performed on a clinical basis in cases strongly suggestive of leptospirosis. Except for rapid tests, all other tests (ELISA, MAT, PCR) are performed in the reference centers. The inclusion of both methods (PCR and MAT) in the diagnosis of leptospirosis is crucial as leptospirosis presents with a biphasic illness. The illness comprises of two distinct clinical phases, which are the leptospiraemia (septicemia) or acute phase and the immune phase. In the leptospiraemia or acute phase, the leptospires that infected the host will be present in the blood and cerebrospinal fluid (CSF) [12, 17]. This septicemia phase with febrile illness characteristics may last from 3 to 10 days [8, 12]. In the second phase (immune phase), antibodies are released into the blood due to the body's immunologic response against the presence of the leptospires. The immune phase usually occurs in the second week and lasts from 4 to 30 days [12]. It coincides with the elimination of the leptospires from the blood and also accompanied by the excretion of leptospires in the urine. Since the actual onset of illness is not clear in many patients, the usage of only one method, either MAT or PCR, may miss cases or lead to misdiagnosis. This study was undertaken to determine whether the combination of both MAT and PCR could increase the number of leptospirosis cases detected in patients clinically suspected for leptospirosis.

## Materials and methods

### Ethics approval

The ethics clearance for this study was obtained from the Medical Research and Ethical Committee (MREC), Ministry of Health Malaysia (NMRR-15-2148-27536). Written informed consent was obtained from all patients who participated in the study.

### Patient recruitment and sampling

Patients clinically suspected for leptospirosis (identified by the attending physician) were recruited from two states in Central Malaysia (Selangor and Perak) according to the guidelines from the Ministry of Health, Malaysia [18]. Patients with acute febrile illness, with history of exposure to water and/or environment possibly contaminated with infected animal urine with any of the following symptoms such as headache, myalgia, arthralgia, meningeal irritation, jaundice, conjunctival suffusion, skin rash, anuria or oliguria, cardiac arrhythmia or failure, hemorrhages in intestines or lungs and gastrointestinal symptoms were recruited. The study was conducted in collaboration with three hospitals (i.e., Hospital Serdang and Hospital Tengku Ampuan Rahimah (HTAR) in Selangor state, and Hospital Teluk Intan in Perak state) from January 2016 to December 2017. Blood specimens for serology (i.e., in a plain tube) and PCR (i.e., in Ethylenediaminetetraacetic acid (EDTA) tubes) were collected from all participating patients upon admission. Upon availability, blood specimens for serological testing were also collected from the recruited patients during discharge and two weeks after discharge. Blood collected in EDTA tube was stored in -40°C freezer for DNA extraction. A total of 165 patients clinically suspected for leptospirosis (Serdang Hospital (n = 59), HTAR (n = 16), Teluk Intan Hospital (n = 90)) were enrolled in this study. From all 165 patients, serum was collected during admission. Among the 165 cases, for 63 (38%) paired sera were available while the remaining patients rejected the invitation for a follow up after discharge or could not be contacted. Blood samples were available for only 75 patients (one of the participating hospitals did not collect blood separately in EDTA tube). MAT was performed for all 165 samples collected during admission and 63 follow up samples. PCR was performed on 75 blood and 90 serum samples (those patients for whom the whole blood was not available for testing). The day post onset of symptoms (DPO) was recorded at the time when specimens were collected from the patients.

### Serology and molecular detection

#### Microscopic agglutination test

For serology, all serum samples were subjected to MAT as described earlier [14]. MAT was performed using a panel of 20 local and international serovars comprising of pathogenic and non-pathogenic leptospires. International panels were obtained from World Health Organization (WHO) Leptospirosis collaborating center, Amsterdam (Australis, Autumnalis, Batavia, Canicola, Celledoni, Grippotyphosa, Hardjoprajitno, Icterohaemorrhagiae, Javanica, Pyrogenes, Tarrasovi, Djasiman, Patoc, and Pomona) while local serovars were obtained from the Institute for Medical Research (IMR), Federal Territory of Kuala Lumpur, Malaysia (IMR LEP 1; saprophyte, IMR LEP 115; saprophyte, IMR LEP 175; saprophyte, IMR LEP 803/11-Copenhageni, IMR LEP 27-Hardjobovis, IMR LEP 22-Lai). Serum samples were diluted to 1:25 with phosphate-buffered solution (PBS) and 50 μl of the diluted sera were used to screen for agglutination against each serovar. Those samples that showed positive agglutination were subjected to serum titration to determine the titer. Samples were considered MAT positive if at least 50% of the leptospires agglutinated with the serum at titer ≥1:400 for single serum specimen or a fourfold rise (seroconversion) in paired samples [15].

#### Molecular detection using PCR

For molecular detection, all blood or serum samples were subjected to DNA extraction (DNAeasy Blood and Tissue kit, Qiagen, German). The 242bp *lipL32* (primers: LipL32-45F: 5′-AAGCATTACCGCTTGTGGTG-3′, LipL32-286R: 5′-GAACTCCCATTTCAGCGATT-3′, probe: LipL32-189P: FAM-5′-AAAGCCAGGACAAGCGCCG-3′-BHQ1) [19] and 547bp 16S rDNA (*rrs*) (outer primers: rrs-outer-F: 5′-CTCAGAACTAACGCTGGCGGCGCG-3′, rrs-outer-R: 5′-GGTTCGTTACTGAGGGTTAAAACCCCC-3′, inner primers: rrs-inner-F: 5′-CTGGCGGCGCGTCTTA-3′, rrs-inner-R: 5′-GTTTTCACACCTGACTTACA-3′) [20] were used as gene targets for molecular detection. DNA extracted from *L*. *interrogans* serovar Copenhageni and nuclease-free water served as positive and negative controls respectively for both PCR assays. The final reaction mixture for *lipL32* qPCR contained 12.5 μL Quantinova master mix (Qiagen, German), 1 μL of 10 pmol of each primer, 0.5 μL of 10 pmol probe and 10 μL of DNA extract from clinical specimens in a final volume of 25 μL. All reactions were performed on Eppendorf MasterCycler® Realplex, with PCR program comprising 8 min at 95°C, followed by 45 cycles of amplification (95°C for 3 s and 58°C for 15 s) and finishing with a cool cycle of 45°C for 90 s. A standard curve was generated for each PCR reaction to determine the copy number of leptospires in each sample.

Nested 16S rDNA gene detection was performed on conventional PCR (Biorad). The first PCR reaction containing 12.5 ul of Econotaq PLUS GREEN 2X master mix, 1 ul of each 10 pmol rrs-outer primers and 10.5 ul extracted DNA was performed using the following condition: initial incubation of 95°C for 2 minutes followed by 40 cycles of 95°C for 10 seconds, 67°C for 15 seconds and 72°C for 30 seconds, and one last cycle at 72°C for 7 minutes. The second PCR reaction containing 12.5 ul of Econtaq PLUS GREEN 2X master mix, 1 ul each 10 pmol of rrs-inner primers, 10.5 ul of first PCR product was performed using the following condition: one cycle of 95°C for 2 minutes; 40 cycles of 95°C for 10 seconds, 57°C for 15 seconds and 72°C for 30 seconds, then one cycle of 72°C for 7 minutes. Amplicons were visualized using 1.4% gel electrophoresis, stained with Gel Red (HealthView Science). A positive PCR result was defined as the visualization of the band at the predicted size (547 bp) of *rrs* gene.

### Statistical analysis

A Chi-square test (χ^2^ test) was performed using the IBM SPPS Statistics 22 to determine if DPO influences the positivity of PCR and MAT. A p-value of less than 0.05 is considered statistically significant.

## Results

**Fig 1:**
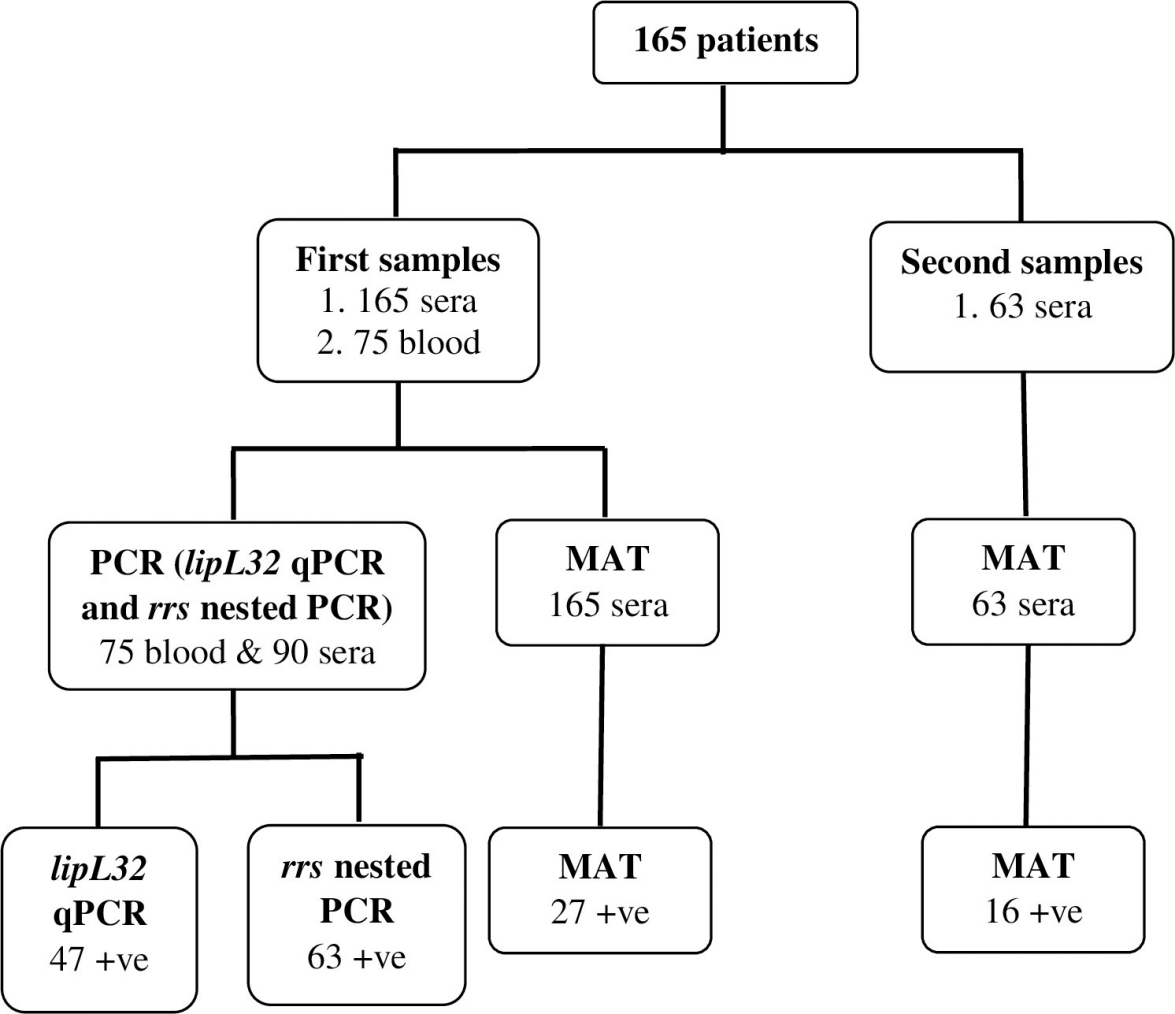
Sampling scheme, diagnostic tests used in this study, and the number of positive samples based on each test.

**Fig 2:**
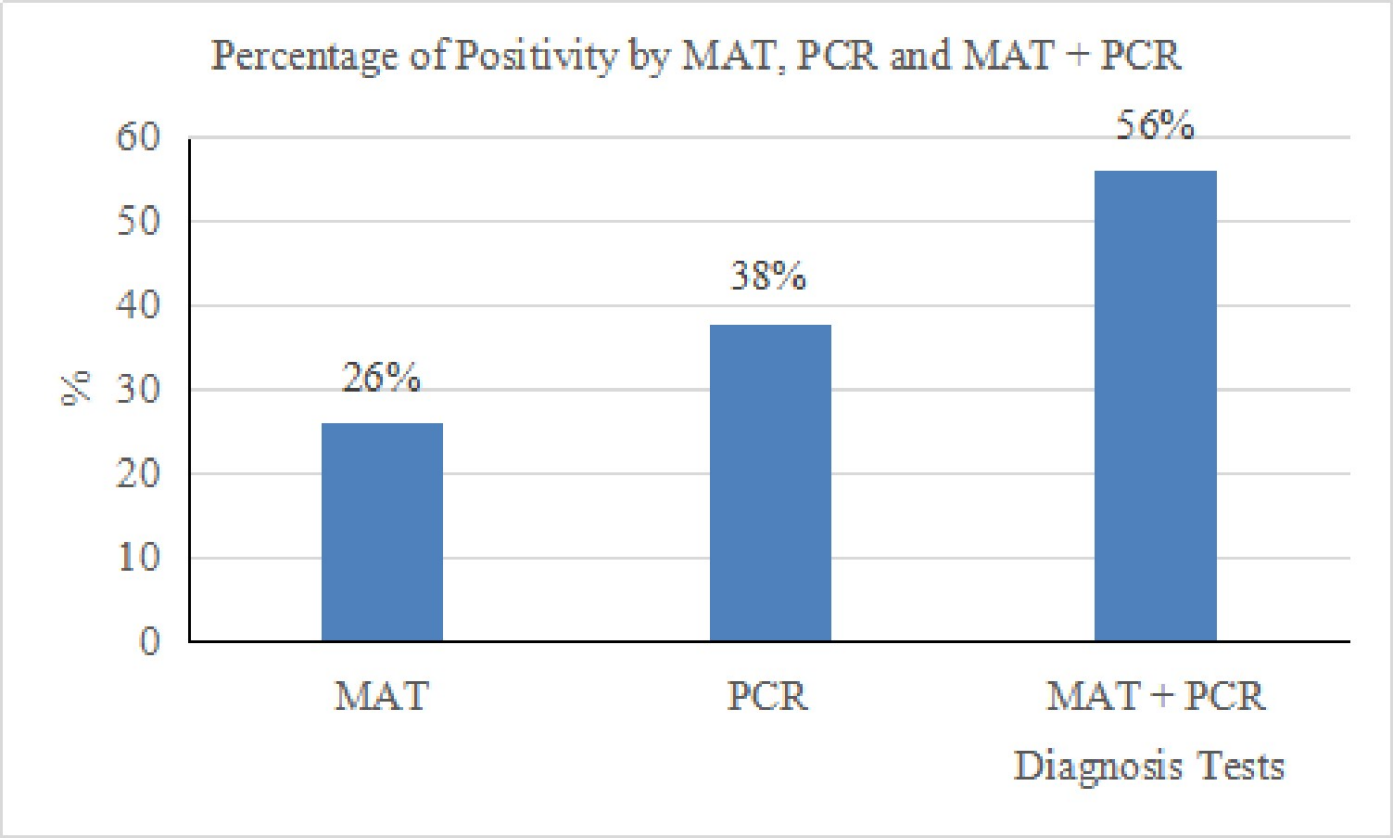
Percentage of positivity MAT, PCR, and MAT+PCR. The detection rate is increased when both MAT and PCR were included in the diagnosis of leptospirosis.

From the 165 patients recruited in this study, 43 (26%) of them were positive by MAT and 63 (38%) by PCR (Table 1). Among all, 14 patients (n = 14; 8%) were positive for both MAT and PCR. From the 43 MAT positive patients, 16 were confirmed as positive based on titer from the second sample (seroconversion) (Fig 1). For molecular detection, among the 165 clinically suspected leptospirosis patients, *lipL32* was detected in 47 (29%) and *rrs* gene in 63 (38%) patients (Table 1 and Fig 1). All samples positive in *lipL32* qPCR were also positive in the *rrs* nested PCR. *LipL32* qPCR showed ct values between 28.96 and 39.67 with copy numbers ranging from 1 to 9.9 x 10^2^ GE/μl. In total, 92 (56%) patients were confirmed positive for leptospirosis either through MAT or PCR and it showed that the positivity increased when both methods were combined (Fig 2). The representative pictures for *lipL32* qPCR output and gel picture for *rrs* gene are provided in S1 File. Since both MAT and PCR are confirmatory methods and there is no gold standard test for leptospirosis diagnosis, these tests (MAT and PCR) were not compared with any standard, hence the sensitivity, specificity, Positive Predictive Value (PPV) and Negative Predictive Value (NPV) were not calculated in this study.

**Table 1:** Number of confirmed leptospirosis patients based on MAT and PCR.

Tests	MAT	PCR			MAT and PCR	Total Positive
		*lipL32*	*rrs*	*lipL32 + rrs*		
No. of positive patients	43/165 (26%)	47/165 (29%)	63/165 (38%)	63/165 (38%)	14/165 (8%)	92/165 (56%)

To determine whether DPO influences the positivity of diagnostic tests, detection by MAT or PCR was calculated for all 228 samples tested (165 first samples + 63 second samples) during the 0–8, 9–15, and ≥16 DPO intervals, as shown in Table 2. Most patients with samples obtained during the first eight days of illnesses were positive by PCR (n = 52/92, 57%). MAT showed higher positivity (n = 17/92, 18%) than PCR (n = 11/92, 12%) between 9 to 15 days of the illness presentations. Only 11 (12%) patients were positive by both methods in the early course of the disease (≤8 days of clinical presentations). The difference in the positivity of PCR in two consecutive DPO intervals (0–8 days and 9–15 days) was significant (p<0.05), indicating that the positivity of PCR (82.5% were positive when tested between 0–8 days, while 17.5% between 9–16 days) is dependent on the days of clinical presentations. Although the positivity of MAT was higher in ≤8 DPO than in ≥9 DPO (58.1% positive in less than 8 days post-onset and 39.5% by 9–16 days and 2.3% after 16 DPO), however, the difference was not significant.

**Table 2:** Number of samples positive in either MAT or PCR, or both MAT and PCR according to the onset of symptoms.

DPO	Total samples tested	PCR only	MAT only	PCR and MAT	No. of positive samples
≤8	154	41	14	11	66
9–15	54	8	14	3	25
≥16	20	-	1	-	1
Total	228	49	29	14	92

*DPO = Days post-onset of symptoms.

## Discussion

The diagnosis of leptospirosis remains challenging mainly due to its non-specific symptoms and the broad spectrum of clinical presentations. Its biphasic nature further complicates the diagnosis as not a single test is adequate for the confirmation of leptospirosis. In this study, PCR showed higher positivity than MAT in ≤8 DPO underscoring its diagnostic sensitivity in the early phase of the illness. The serology-based MAT is comprehensive only when the paired serum is available, which is the major challenge as most patients were discharged in one to three days and did not cooperate for a follow-up sample after discharge either due to logistical issue or not contactable (*i*.*e*. in the case of patients such as foreign labours). Most of the leptospirosis positive patients in the present study had daily waged occupation. Hence taking one day leave from work will incur a loss of salary and transport charges which restricted them for the follow-up sample. This limited us from getting paired samples for many of the patients.

Based on the PCR approach, the number of samples positive by *rrs* was higher than the *lipL32* gene. This might be due to the higher copy number of the *rrs* gene in the *Leptospira* genome (two copies of *the rrs* gene and only one copy of the *lipL32* gene) [21–23]. This finding is also supported by a previous study that reported *rrs* assay (qPCR) showed higher sensitivity compared to *lipL32* (qPCR) (56% versus 43%) when tested on blood samples from leptospirosis patients [22]. The above finding reemphasizes that testing for more than one gene target for the detection of leptospires could improve the efficacy of the molecular-based diagnosis. The different PCR methods may also influence the positivity rate between the two methods. For leptospirosis diagnosis, TaqMan based qPCR is routinely performed than the classical PCR due to its short turnover time and detection of low copy numbers of DNA. In the present study, we found higher leptospires detection by *rrs* nested PCR. The nested PCR is a two successive PCR runs of reaction, in which the second set is intended to amplify a secondary target within the first run product. Therefore, this allows running more cycles (more amplification) for a low number of target DNA amplified in the first PCR round. Since both tests (qPCR and nested PCR) were not compared using the same target gene, it is not appropriate to recommend which test is more sensitive. However, a previous study performed by Kim and colleagues showed that nested PCR had slightly higher sensitivity than the qPCR (85.4% vs 82.9%) [24]. A highly sensitive multiplex PCR that could detect both *lipL32* and *rrs* gene targets in a single reaction will be more robust, fast, economic, and time-saving early diagnosis molecular test for leptospirosis.

It is well understood that, in the biphasic nature of leptospirosis, leptospires will be present in the blood during the first week of clinical presentations and will be eliminated as the level of antibody arise in the second phase of the disease, typically at the second week of illnesses [12]. This notion is supported through this study in which a significant correlation (p<0.05) was found between DPO and detection rate; among the 63 PCR positive patients, 52 presented with clinical manifestations within the eight days of disease presentations through their blood samples. PCR showed higher positivity during the first eight days of illness (p<0.05), however, it was negative in 14 patients who were MAT positive. Although the DPO was calculated based on history taking from patients, the actual exposure to the organism is largely unknown. Moreover, even though seroconversion commonly occurs on the second week and up to the fourth week after infection [25], it may also occur as early as in 4 to 7 days [11, 26, 27], thereby reduces the leptospiral density in the blood to an undetectable level. On the other hand, MAT failed to detect 41 positive cases that were PCR confirmed. There were only 11 patients confirmed for leptospirosis by both methods in the first eight days of clinical presentations. It was also shown in this study that eight patients, whose samples obtained during the second week of disease presentations were PCR positive but negative by MAT. This implied that though DPO is significantly associated with the diagnostic test, the use of a single test or selection of diagnostic tests based on the number of days of illnesses might not be applicable in the diagnosis of leptospirosis. Thus, the data obtained from the present study strongly emphasize the need to include both PCR and MAT for every first sample when the patient is suspected for leptospirosis to increase the early diagnostic efficacy and to avoid misdiagnosis regardless of DPO. This is in agreement with a previous study that has also reported the usefulness of combined methods (PCR and MAT) in the diagnosis of leptospirosis [28].

## Conclusions

The combination of both PCR and MAT is important for early diagnosis of leptospirosis as it not only increases the detection rate but also avoids misdiagnosis, hence facilitating the correct management of the patients. We would also like to emphasize the importance of using more than one marker in PCR and developing a highly sensitive multiplex PCR for improving the detection rates.

## Supporting information

S1 File Figures for *lipL32* qPCR output and *rrs* nested PCR. (DOC)
