# Supplementary material for: Combined PCR and MAT improves the early diagnosis of the biphasic illness leptospirosis
Source: PLoS One. 2020 Sep 11;15(9):e0239069. doi: 10.1371/journal.pone.0239069 (PMC7485768; doi:10.1371/journal.pone.0239069)
Supplement: S1 File — (DOC) [file pone.0239069.s001.doc]

**Supplementary File 1.** Figures for *lipL32* qPCR output and *rrs* nested PCR.

NC


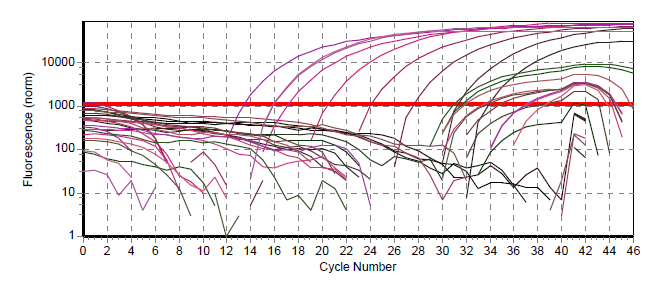

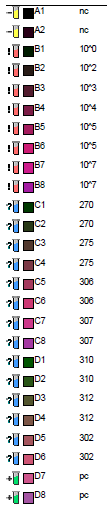


TI315

TI306

TI306

TI276

TI276

TI270

TI270

10^7

10^6

10^5

10^4

10^3

10^2

NC

10^1

10^0

TI315

PC

PC

TI302

TI302

TI313

TI313

TI243

TI243

**SFig 1. Representative qPCR amplification plot for *lipL32* gene.** In this qPCR output, amplification below ct values of 30 represents four standard samples of known concentration (10^4-10^7) diluted in 10-fold serial dilutions and positive control (pc). Unknown samples (samples labelled as TI276, TI315, TI243, TI302) that were detected positive had ct values above 30. NC represents negative samples and did not show any amplification. Every samples were ran in duplicates. Most samples detected using *lipL32* qPCR had ct values between 28.96 and 39.67.


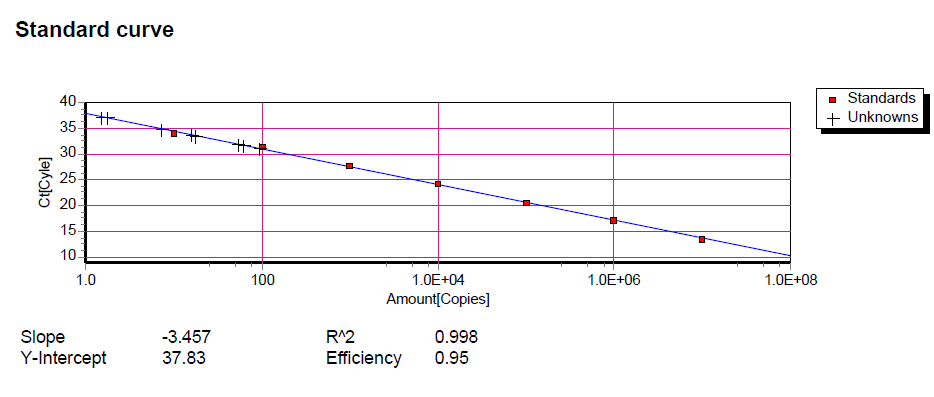


**SFig 2. Standard curve for Fig 1 qPCR output.** Samples (unknown) have ct value above 30.


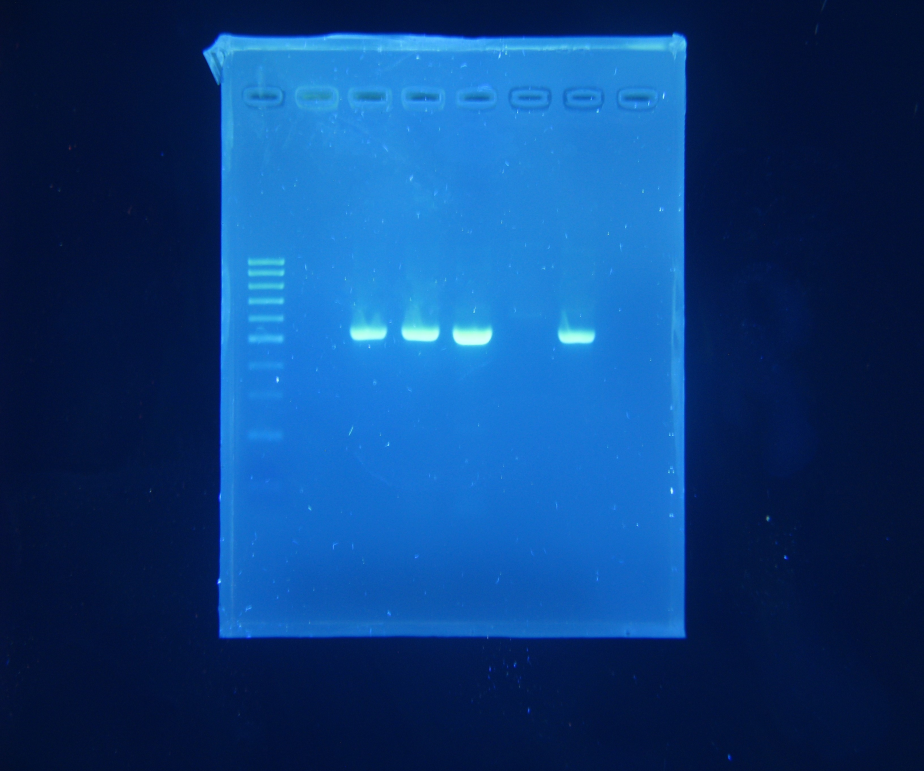


100

200

300

400

500

600

700

800

900

1000

Base pairs

SP35

SP32

SP31

SP28

DNA

ladder

SP27

NC

PC

**SFig 3**. **Representative gel picture for *rrs* gene (547 bp).** NC indicates negative control, PC as positive control and SP27, SP28, SP31, SP32 and SP35 as samples. SP27, SP28 and SP32 represent positive samples while SP31 and SP35 represent *rrs* negative samples.
